# The Tick Microbiome: The “Other Bacterial Players” in Tick Biocontrol

**DOI:** 10.3390/microorganisms12122451

**Published:** 2024-11-28

**Authors:** Paulina Maldonado-Ruiz

**Affiliations:** Department of Entomology, College of Agriculture, Life and Environmental Sciences, University of Arizona, Tucson, AZ 85719, USA; lpmaldonado@arizona.edu; Tel.: +1-520-621-3799

**Keywords:** tick microbiome, bacillus, bacillus thuringiensis, tick biocontrol, amblyomma americanum, *Ixodes* spp.

## Abstract

Hard ticks (family Ixodidae) are one of the most predominant arthropod disease vectors worldwide, second only to mosquitoes. In addition to harboring animal and human pathogens, ticks are known to carry a microbial community constituted of non-pathogenic organisms, which includes maternally inherited intracellular endosymbionts and other environmentally acquired extracellular microorganisms. These microbial communities, which include bacteria, viruses, protozoans, and fungi—with often commensal, mutualistic, or parasitic associations with the tick—comprise the tick microbiome, bacteria being the most studied community. Many bacterial taxa frequently reported in ticks include soil, plant, and animal-associated microbes, suggesting many are environmentally acquired, including members with known entomopathogenic potential, such as *Bacillus thuringiensis*, *Bacillus* spp., and *Pseudomonas* spp. It has been reported that microbial community composition can impact pathogen persistence, dissemination, and fitness in ticks. In the United States, *Ixodes scapularis* (northeast) and *I. pacificus* (west) are the predominant vectors of *Borrelia burgdorferi*, the causal agent of Lyme disease. *Amblyomma americanum* is another important tick vector in the U.S. and is becoming an increasing concern as it is the leading cause of alpha-gal syndrome (AGS, or red meat allergy). This condition is caused by tick bites containing the galactose alpha 1,3 galactose (alpha-gal) epitope in their saliva. In this paper, we present a summary of the tick microbiome, including the endosymbiotic bacteria and the environmentally acquired (here referred to as the *non-endosymbiotic* community). We will focus on the *non-endosymbiotic* bacteria from *Ixodes* spp. and *Amblyomma americanum* and discuss their potential for novel biocontrol strategies.

## 1. Introduction

Many hematophagous arthropods are important disease vectors, as they carry and transmit pathogens that cause disease worldwide. In addition to pathogens, it is known that these arthropods also harbor a microbiome of non-pathogenic symbiotic bacteria [1,2]. These symbiotic relationships (arthropod–microorganism) can be parasitic, commensal, or mutualistic [3]. The arthropod–endosymbiont relationship is an excellent example of an obligate mutualistic symbiotic association, where both organisms (the vector and the intracellular microorganisms/endosymbiont) are often physiologically dependent on each other [4]. Arthropod endosymbionts have been extensively reported in obligate blood-feeders such as ticks, tsetse flies, and bed bugs [2,3,5], possibly due to their specialization to a nutrient-deficient food source, as they have been shown to provide nutritional benefits to the arthropod, and due to immune system development, which can impact their overall fitness [6,7,8]. One example is the *Coxiella*-like endosymbiont (CLE), which has been suggested to aid in synthesizing biotin, folate, and other vitamins from the B complex in the lone star tick (*Amblyomma americanum*) [9], while rickettsial endosymbionts have been proposed to provide nutrients to the black-legged tick (*Ixodes scapularis*) and the western black-legged tick (*Ixodes pacificus*) [10]. This endosymbiont–arthropod relationship is likely the result of a long evolutionary association between the arthropod host and the bacteria, potentially including the acquisition of bacteria and/or bacterial origin genes through horizontal gene transfer [11]. Intracellular endosymbionts comprise the majority of the bacterial abundance in ticks; nonetheless, non-endosymbiont communities have been found within the tick gut. However, it is yet unknown if these communities play a role in tick vector competence and development or whether they are transient or established bacterial populations.

In this paper, we present a comprehensive summary of the tick microbiome, including the maternally inherited intracellular (endosymbiotic) bacteria and the non-endosymbiotic bacterial community. We will focus on the non-endosymbiotic bacterial members (“the other bacterial players”) from *Ixodes* spp. and *A. americanum* and their relevance for approaches for tick biocontrol. This review includes illustrations from our previous work on the microbiome of *A. americanum* and our most recent findings in *A. americanum* and *Bacillus* spp. interactions.

## 2. The Tick Microbiome

Ticks are ectoparasitic arachnids that belong to the order Ixodida, which includes three families: *Ixodidae* (hard ticks), *Argasidae* (soft ticks), and *Nuttalliellidae* (comprised of only one genus) [12]. Hard ticks (*Ixodidae*) are among the most important arthropod vectors of human and animal pathogens in the United States and worldwide [13,14,15]. They are obligate blood-feeding ectoparasites that carry a wide range of pathogens, such as bacteria, viruses, fungi, and protozoans [15], which can transmit disease to humans and other animals, causing a significant economic burden to healthcare systems worldwide [16,17]. The expansion in tick geographical distribution and the significant increase in tick-borne illness has prompted research to understand, prevent, and control tick-borne disease. This includes the understanding and characterization of the tick microbiome to elucidate their contribution to pathogen acquisition and disease transmission. While the role of the tick microbiome is still poorly understood, it has been shown to impact tick vector competence and overall tick fitness. Most tick microbiome studies have focused on bacterial characterization, primarily capturing tick endosymbionts, while the non-endosymbiotic bacteria have been identified in ticks with lower prevalence and abundance. It has been reported that endosymbionts, such as *Coxiella* sp., *Francisella* sp., *Rickettsia* sp., and *Midichloria mitochondrii*, constitute a significant part of the tick microbiome in different tick species [18,19,20,21,22]. These bacterial communities are often intracellular, maternally inherited bacteria with reduced genomes compared to the bacterial pathogenic species from the same genera; thus, they are suggested to have evolved from a pathogenic ancestor [23,24]; however, their pathogenic potential remains unknown. Nonetheless, the reduced symbiont genome implies that the long mutualistic association allowed for the retention of only the genes essential for survival within the host cell. While nutrient synthesis has been suggested as a role of the endosymbionts in ticks, little is known about the non-endosymbiotic bacterial community, which includes the non-pathogenic, extracellular, and environmentally acquired bacteria.

## 3. Beyond Tick Endosymbionts

**Figure 1:**
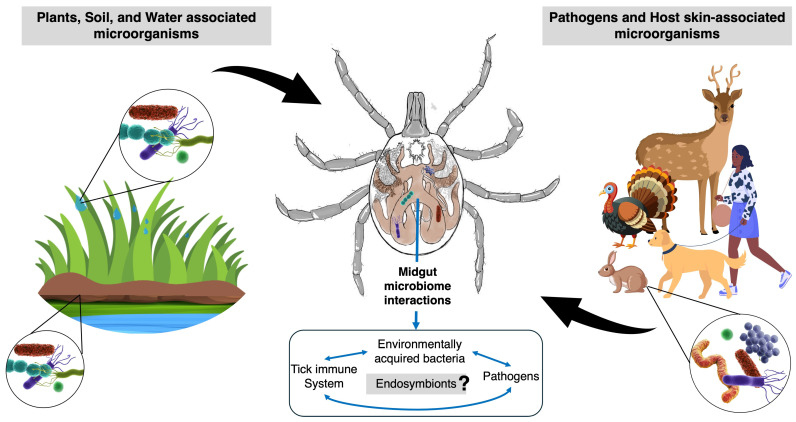
The tick microbiome and its environment. In addition to maternally inherited endosymbionts, the tick microbiome comprises environmentally acquired microorganisms from the soil, plants, and water droplets (**left**), while pathogens and the host’s skin-associated microorganisms are acquired through feeding (**right**). These microbial communities interact with the tick midgut immune system. It is yet unknown to what extent the acquired organisms interact with the tick endosymbionts. The illustration was created and edited using Adobe Illustrator “adobe.com/products/illustrator”; 2019; (accessed on 27 September 2024), Adobe Sketch “www.adobe.com” (accessed on 10 October 2024), Adobe Stock “https://stock.adobe.com” (accessed 10 October 2024), and PowerPoint.

Beyond the maternally inherited microbiota, it has been suggested that ticks have limited opportunity to acquire a diverse microbiome due to their obligate blood-feeding nature, thus limiting bacterial uptake from their microhabitats (plant and soil) and their feeding behaviors (animal host skin) (Figure 1). Studies on the prevalence of non-endosymbiotic bacteria have revealed that ticks do not harbor a core microbiome [25,26,27]. Nonetheless, different bacterial taxa that include typical soil- and plant-associated bacteria, such as *Pseudomonas* spp., *Bacillus* spp., and *Enterobacteriaceae*, have been frequently reported in ticks [26], suggesting they are environmentally acquired [1,25,28]. We previously reported that the lone star tick (*A. americanum*) can uptake water-containing bacteria voluntarily in a laboratory setting [29], which had been previously observed in the field in other tick species larvae [30,31]. These findings support that the bacterial diversity seen in unfed field-collected *A. americanum* may be attributed to the ingestion of water-containing bacteria from the tick microhabitats. Interestingly, in the Lyme disease vector (*I. scapularis*), this “voluntary water-drinking” behavior was only seen in 5% of the ticks in a laboratory setting [32], suggesting that direct ingestion from the environment may be less likely, yet however still a potential form of bacterial uptake (Figure 1). It is yet unclear if these non-endosymbiotic bacterial communities are transient or an intrinsic component of the tick microbiome [33]. Nonetheless, they are suggested to play a role in pathogen persistence in the tick vector [34,35,36]. It is important to highlight that while most studies characterize the microbial community as a “snap-shot” in time (primarily in the unfed stage), it has been observed that the microbiome’s community composition and abundance change with temperature and during blood-feeding [37,38,39].

While the relative abundance of certain bacterial taxa is partially influenced by blood-feeding, the overall species richness is maintained [40]. This implies that the same bacterial taxa are present, while the representative units (sequencing reads) appear lower due to the proliferation/replication of other taxa during feeding. Thus, when assessing comparisons on the tick microbiome across studies, it is important to consider the sampling methods, tick feeding status, tick stage, and sequencing technology used in order to make accurate inferences.

## 4. Tick Microbial Interactions: Endosymbionts and Non-Endosymbionts

Due to their intracellular nature, it is speculated that the tick endosymbiotic community may indirectly interact with the environmentally acquired microorganisms. While it is suggested that interactions between non-endosymbiotic (pathogenic and non-pathogenic) and endosymbiotic bacteria are bidirectional, we have yet to uncover the full scope of these potential interactions (Figure 1). While most studies evaluating the role of the tick microbiome are focused on bacterial communities, it is important to note that these non-pathogenic and pathogenic environmentally acquired microorganisms also include viruses, fungi, and protozoans, which interact with each other and with the tick immune system, eliciting basal immune activity, which prevents microorganism proliferation [41]. New sequencing technologies and pipelines have allowed the characterization of the tick viral [42] and fungal communities [43], which also constitute a part of the tick microbiome. More studies on viral and fungal tick communities are needed to elucidate these microbe–microbe interactions and their potential roles in maintaining tick microbial homeostasis and/or pathogen persistence.

## 5. The Microbiome of *Ixodes* spp. (*Ixodes scapularis*, *I. pacificus* and *I. ricinus*)

The genus *Ixodes* contains the most important human disease tick vector species worldwide, as they are vectors of *Borrelia burgdorferi*, the causal agent of Lyme disease [44]. In the United States, the main vectors of *B. burgdorferi* are *I. scapularis* and *I. pacificus*, while *I. ricinus* is the primary vector in Europe [45]. It has been reported that the predominant endosymbiont of *I. ricinus* is *Midichloria mitocondrii* [21], which has been found in different tick tissues of the unfed and partially fed tick, such as ovaries, trachea, malpighian tubules, midgut, and salivary glands [22,46]. This tissue tropism allows for the speculation of different functional roles of the endosymbiont across tissues [46]. In *I. scapularis* and *I. pacificus, Rickettsia* spp. Has been reported with an overall high abundance across tick life stages [47,48]. The roles of the *Ixodes* spp. Endosymbionts are suggested to be primarily nutrient production and involvement in tick development [49,50]. While a high abundance of *Rickettsia* spp. has been reported in certain *I. scapularis* geographic populations [51], bacterial members of Enterobacteriaceae, which are typically soil and host-associated bacteria (primarily extracellular within the tick), have been reported to dominate in other *I. scapularis* populations (Table 1) [51,52]. Interestingly, an earlier study on the isolation of aerobic bacteria from field-collected adult and nymph *I. scapularis* reported 63 different bacterial isolates, many of which were *Bacillus* spp., including *Bacillus thuringiensis* (*Bt*), which was frequently isolated from both adult and nymph ticks (Table 1) [53]. Furthermore, an integrated study using metatranscriptomics and metaproteomics for the characterization of the *I. ricinus* microbiome reported many environmentally associated bacterial members and other commensals, such as *Acinetobacter* sp., *Pseudomonas* sp., and many Enterobacteriaceae, in addition to the frequently reported endosymbionts [54]. These non-endosymbiont bacterial members have been suggested to play a role in pathogen colonization. It was demonstrated that microbial dysbiosis in *I. scapularis* impaired *B. burgdorferi* colonization [55]. While the mechanisms are yet unknown, it is suggested that the natural microbiota of *I. scapularis* maintain a favorable environment, providing structural and functional integrity in the midgut that allows pathogen invasion [55,56].

It is important to note that the microbial community composition varies according to geographical location, feeding status, stage, and sex. Significant differences in bacterial community composition have been reported in *I. scapularis* males when compared to females, with unfed males harboring *Pseudomonas*, *Brevibacterium*, *Bradyrhizobium*, and *Sphingomonas* in high relative abundance (15 to 30%), while females show less than 5% of these taxa (Table 1) [39]. Furthermore, high temperatures have been shown to drop bacterial community complexity in *I. scapularis*, as ticks incubated at 37 °C for at least 5 days showed a decrease in *Pseudomonas* and an increase in *Brevibacterium* [39].

## 6. The Microbiome of the Lone Star Tick, *Amblyomma americanum*

The lone star tick (*A. americanum*) is commonly distributed in the Midwest and Eastern United States [61]; however, its geographical distribution is rapidly expanding [62,63,64]. The lone star tick is a vector of several pathogens that cause disease in humans and companion animals, such as *Francisella tularensis*, *Ehrlichia chaffeensis*, *E. ewingii*, and Heartland virus [65,66,67,68]. This tick has also been identified as the main sensitizer for alpha-gal syndrome (AGS, or red meat allergy) in humans, which is an allergic reaction triggered by the enhanced production of IgE antibodies against the galactose alpha 1, 3 galactose alpha-gal (alpha-gal or aGal) epitope commonly found in mammalian meat and dairy products [69]. The main sensitizers for AGS are bites of *A. americanum* that inject the alpha-gal carbohydrate contained in their saliva [70,71]. This lifelong condition is a growing concern for the healthcare system as a significant increase in AGS cases has been reported in recent years [72]. While the alpha-gal in tick saliva is believed to be produced by the tick [73], the mechanisms remain unknown. Nonetheless, the tick bacterial microbiome cannot be excluded as a potential contributor to alpha-gal synthesis in ticks [74].

Most studies on the microbiome of *A. americanum* have reported that 80% to over 90% of the microbiome comprises intracellular endosymbionts, primarily *Coxiella*-like and *Francisella*-like endosymbionts, while *Rickettsia* is reported with a lower abundance [20,60,75,76,77]. The *Coxiella*-like endosymbiont has been shown to contain genes from six of the eight cofactor biosynthesis pathways, suggesting potential for vitamin production within the tick [9]. Antibiotic treatment of *A. americanum* engorged nymphs and adults exhibited lower weights, longer oviposition time in the adults, and a lower number of viable offspring [78]. In this study, the fitness estimators were associated with a lower number of *Coxiella* sp. but not *Rickettsia* sp., although overall *Rickettsia* sp. levels were also reduced. These findings suggest *Coxiella* sp. is essential for *A. americanum* fitness; however, the overall bacterial community composition was not evaluated. Thus, the impacts of other non-endosymbiont bacterial members on tick fitness are yet unknown. A 2016 study aimed at elucidating the microbiome’s potential contribution to pathogen acquisition reported no significant differences in the *A. americanum* microbiome between ticks infected with *Ehrlichia* and *Ehrlichia*-uninfected ticks [59]. Nonetheless, specific differences between infected and uninfected ticks were observed in certain Operational Taxonomic Units (OTUs) of uninfected ticks, where members of Proteobacteria, Actinobacteria, and Bacteroidetes were overrepresented when compared to the infected ticks [59]. Furthermore, many non-endosymbiotic environmentally associated bacteria have been frequently reported and isolated from the internal tissues of *A. americanum* using culture-dependent and culture-independent methods (Table 1) [25,40,58,59,60].

## 7. The Non-Endosymbiotic Bacteria and Culture-Dependent Isolation Methods

Using different bacterial sequencing approaches, earlier studies have shown great microbial diversity in *A. americanum*, reporting several hundreds of operational taxonomic units (OTUs) with high alpha diversity, at least 99 bacterial families, and over 100 genera [37,58,59]. Sequencing of the 16S rDNA gene has been the standard method for microbiome studies. Nonetheless, the use of different sample processing methods, such as tick surface sterilization, amplification of different hypervariable regions of the 16S gene, and the use of different sequencing platforms, makes comparisons across tick microbiome studies challenging [52,79,80]. It is important to note that while these studies provide significant insights into the tick microbiome composition, they do not provide information on the viability of the bacterial members, as any bacterial DNA present in the sample will be amplified. Moreover, most culture-independent tick microbiome studies report bacterial communities that are culturable using broad-spectrum media such as *Bacillus* spp., *Pseudomonas* spp., *Micrococcus* spp., *Staphylococcus* spp., and many Enterobacteriaceae (Table 1) [25,57,59,60]. Thus, culture-independent studies coupled with a culture-dependent approach are needed to determine the abundance of viable (live) bacterial taxa. Recent studies comparing culture-dependent and culture-independent approaches for bacterial abundance assessment showed low abundance and diversity of culturable extracellular bacteria in ticks [25,57]. We previously suggested that the overrepresentation of endosymbionts in ticks likely underestimates the abundance of extracellular bacteria, as several culturable bacteria isolated using broad-spectrum media were not captured with the sequencing approach [25]. Furthermore, the characterization and isolation of prevalent bacteria in the tick gut using culturable methods allows for easy isolation for future studies on the manipulation of the gut bacterial community to evaluate their effects on tick vector competence.

## 8. The (Un)Favorable Tick Midgut Environment for the Microbiota

The tick midgut is the first barrier that the incoming pathogens have to overcome. Thus, it is likely the most important tissue for pathogen survival and proliferation [81]. Due to their blood-digestion strategy (intracellular lysosome-like vesicles), the ingested blood is accumulated in their numerous branching diverticula (caeca), which prevents the incoming bacteria, including pathogens, from being in contact with digestive proteases [82]. This also provides an ideal environment for pathogen proliferation; thus, ticks possess highly efficient gut epithelial immune mechanisms that control the proliferation of microorganisms and maintain microbial homeostasis [81]. These immune mechanisms include the production of anti-microbial peptides (AMPs) and other effector molecules through immunodeficiency (IMD) and JAK-STAT pathways [83,84]. While the IMD pathway appears to be present in *I. scapularis*, important components of this pathway have not been found in ticks [85]. Furthermore, it is suggested that ticks maintain redox homeostasis in their midgut by the production of reactive oxygen species (ROS) [81]. It has been shown in *Drosophila melanogaster* that dual oxidases (Duox) decrease microorganisms in the gut to prevent bacterial proliferation and thus maintain gut homeostasis through ROS production [86,87]. However, more studies on the direct and indirect impacts of ROS in pathogen invasion are needed to understand their role in tick vector competence and in maintaining gut microbial homeostasis.

In addition to gut immune responses, ticks also contain tissue barriers that prevent microbe internalization and impact pathogen dissemination, such as the peritrophic membrane (PM, or peritrophic matrix) and the dityrosine network (DTN) [56,88,89]. In arthropods, the PM is primarily composed of chitin, proteins, and carbohydrates, and it functions as a selectively permeable molecular membrane that serves as a mechanical barrier for pathogens, abrasive components in the blood, and toxins [89,90]. In *I. scapularis*, the vector of *B. burgdorferi*, a disruption in the PM resulted in borreliacidal responses in the tick gut [88], while the impairment of DTN formation resulted in a reduction of *B. burgdorferi* [56]. The formation of DTN in *I. scapularis* has been attributed to Duox, which was evidenced by the RNAi-Duox knockdown showing an impaired DTN. Interestingly, high expression of Duox in the tick gut coincided with gut microbe replication, DTN impairment, and a reduced abundance of *B. burgdorferi*. Thus, it is unknown if the overexpression of Duox also contributed to pathogen reduction through the production of ROS [56]. Nonetheless, studies on Duox and other peroxidases are needed to elucidate the mechanisms for microbial homeostasis and pathogen persistence in the tick gut.

## 9. Use of *Bacillus* spp. As a Tick Biocontrol Strategy

In our previous work pairing culture-dependent and culture-independent approaches for the characterization and isolation of culturable bacteria of field-collected *A. americanum*, we isolated live *Bacillus* spp. strains from questing and moribund ticks [25]. The microbiomes of unfed questing *A. americanum* showed low culturable bacterial frequency (40%) and primarily included plant- and soil-associated bacterial taxa, such as *Bacillus thuringiensis* (*Bt*), *Bacillus* spp., *Micrococcus* spp., and *Pseudomonas* spp. While few *Pseudomonas* species have been shown to exhibit entomopathogenic potential [91,92], extensive studies report the entomopathogenic effects of *Bt* in over 30 serovars on many arthropods, some of which show toxicity to ticks (Table 2) [93,94]. Studies have shown that *Bt* collected from the field, along with various laboratory strains of *Bt*, can cause delayed tick toxicity (more than 15 days after *Bt* exposure) in different tick species [95,96,97]. This effect was observed when ticks were immersed in highly concentrated *Bt* inocula (up to 2 × 10^9^ CFU/mL) or solutions of *Bt* toxins [98,99]. These studies show that some *Bt* strains can cause mortality to *I. scapularis*, *I. ricinus*, and *Dermacentor reticulatus* (Table 2).

Ixodidae ticks are subdivided into the following two groups: Prostriata, containing one genus (Ixodes), and Metastriata, containing 11 genera, including *Amblyomma*, *Dermacentor, Haemaphysalis*, and *Rhipicephalus* [12]. It has been previously reported that metastriate ticks can directly uptake (drink) water [29,30,31], while prostriate ticks generally show avoidance of water, with only 5% of *I. scapularis* showing “drinking-like” behavior [32]. We previously developed a voluntary tick-feeding approach, which can be used to deliver bacteria to the ticks effectively, and these bacteria can be later recovered from the tick midgut [29]. This voluntary feeding approach consisted of providing a droplet of *Bacillus*-containing water, including *Bt* (a mixture of live—germinating—cells and spores), and allowing the ticks to walk towards the droplet voluntarily. Using this voluntary feeding approach to deliver live *Bt* strains to adult and nymph *A. americanum* ticks, we observed between 30% to 68% mortality within 7 days (Table 2) [29]. Interestingly, our study feeding *Bt* israelensis toxins Cry4B and Cry11A showed no toxic activity to *A. americanum* [29], and the mechanisms of *Bt* toxicity in ticks remain unknown. Using our previously described voluntary feeding method, in our preliminary study, we observed 60% mortality in adult female *A. americanum* after three consecutive daily feeding exposures with *Bt* kurstaki at a dose of 1.6 × 10^7^ CFU/mL (Table 2) (this paper).

Bacterial colony-forming units (CFUs) found in the midgut homogenates of dead individuals from the *Bt* kurstaki treatment showed that the ticks contained 100–10,000 CFUs/tick at the time of death, while surviving individuals contained 0–1000 CFUs/tick. We have previously described that an adult female tick can ingest 0.55 ± 0.06 µL of liquid water [29]. Thus, we estimated that these bacterial numbers (~10,000 CFU) were similar to the bacterial cells ingested by the ticks on day one. In contrast, other Gram (+) and Gram (−) bacteria, such as *Staphylococcus epidermidis* and *Escherichia coli*, delivered to the tick through voluntary and capillary drinking, were rapidly eliminated from the tick gut (*not published*). Our findings on the presence of *Bt* kurstaki in dying ticks suggest that mortality may be caused by the effects of *Bt* persistence in the guts and that mortality may be dose-dependent.

## 10. *Bacillus* Toxicity Mechanisms in the Tick Gut Are Unknown

Tick midgut anatomy and structural changes have been studied during the course of molting and blood feeding in some tick species, including *Amblyomma cajennense*, *I. scapularis*, and *I. ricinus* [100,101,102]. However, little is known about the cellular and subcellular changes in response to bacterial infection processes. It has been well documented that the mode of action of the entomopathogenic bacterium *Bt* is through the release and activation of Cry toxins, which act on a number of receptors that are exclusive to many arthropods [103,104]. It is reported that *Bt* and its toxins induce severe cytotoxicity to the midgut of various arthropods, including caterpillar moths and *D. melanogaster*, which causes midgut cellular disorganization, microvillus degeneration, cell fragmentation, peritrophic membrane rupture, and cell vacuolization [103,104,105,106].

In our preliminary work using voluntary tick feeding to deliver *Bt* live cells and spores, we recovered *Bt* from dead ticks in the same numbers as they had initially ingested. Furthermore, we observed that other *Bacillus* spp. (*B. flexus*—from a tick-lab colony—and *Bt* israelensis) exerted varied levels of tick-killing potential, with the surviving individuals showing highly varied CFU numbers after 8 days (0–1000 CFUs/tick). It is unknown if the *Bacillus* spp. toxicity observed in our preliminary trials with *A. americanum* is mediated through toxins or induced by bacterial cell infection. However, the delayed mortality suggests the cause may be a bacterial infection and/or microbial dysbiosis in the tick gut. Nonetheless, the mode of action of *Bacillus* spp. toxicity in ticks is still a vastly understudied area. Further evaluation of the midgut histopathology during *Bacillus* spp. invasion will help to elucidate the types of structural changes in the midgut epithelium and aid in the understanding of whether toxicity occurs through bacterial internalization to midgut cells or severe cellular vacuolization and membrane rupture, like the case of toxin-mediated toxicity.

## 11. *Bacillus* spp. As the Trojan Horse in Tick Control: A Paratransgenesis Approach

Paratransgenesis is an innovative concept that aims to control the vector or reduce its vectorial capacity by using genetically modified bacterial symbionts [107,108]. Modern technologies using direct genetic editing of the vector have also been employed, namely the use of refractory (resistant to infection) mosquitoes to replace vector populations and the release of mosquitoes carrying a lethal gene to reduce mosquito populations [109,110]. However, finding an effective drive mechanism and developing a stable mosquito germ line can be challenging [111,112,113]. Genome editing using CRISPR/Cas9 has been conducted in *I. scapularis* [114]. However, this technology is still in the development stage. An indirect approach employing genetic modification of endosymbiotic bacteria (paratrasgenesis) has been proposed as a potential strategy for vector-borne disease control [108]. For a paratransgenic approach, one of the main requirements is to identify a microorganism that can successfully colonize the vector without affecting its overall fitness. Symbionts from the Chagas disease vector (*Rhodnius prolixus*) have been transformed using *R. rhodnii* and used as vehicles for the introduction of foreign genes into the disease vector [107]. These transformed symbionts have been successfully introduced in the Chagas disease vector and recovered after molts. In the sleeping sickness (human African trypanosomiasis) vector (*Glossina* spp. or tsetse flies), a similar genetic transformation approach has been applied using the modified *Sodalis* symbiont to repopulate the vector, where they express trypanocidal products with the potential to block parasite development [115,116,117]. This is a promising strategy for the introduction of genes that could prevent pathogen colonization in the arthropod vector.

Paratransgenesis for vector-borne disease control may also be applicable to ticks. In our preliminary study feeding *Bt* kurstaki, *Bt* israelensis, and *B. flexus* to *A. americanum* ticks, we recovered all *Bacillus* strains from the surviving individuals on day 8 after bacterial ingestion. This suggests that the mortality may be dose-dependent and that *Bacillus* may persist in the tick gut, suggesting the potential for establishment. Furthermore, the use of easily culturable bacterial strains and commercially available *Bacillus* strains, such as *Bacillus subtilis,* with mature genetic tools offers a vast number of opportunities for metabolic engineering. In addition to vector and vector-disease control, bacterial colonization/establishment can have ample applications in tick research, such as the elucidation of the intricate tick immune mechanisms in the gut, non-endosymbiotic bacteria’s role in pathogen invasion, and the physiology of tick microbial homeostasis.

## 12. Concluding Remarks

Intracellular endosymbionts, such as *Coxiella*-like, *Rickettsia* spp., *Francisella*-like, and *Midichloria mitochindrii,* comprise the majority of the bacterial abundance in adult and nymph *Ixodes* spp. and *A. americanum* ticks. However, non-endosymbiont bacterial communities have been frequently reported in the whole bodies of ticks and the midgut. It is hypothesized that non-endosymbiotic bacterial communities play a role in tick vector competence and development and are mainly acquired from tick microhabitats, including the animal host. However, whether they are transient or established bacterial populations still remains to be elucidated. Nevertheless, they are suggested to play a role in maintaining tick midgut homeostasis, which may indirectly impact pathogen persistence. Many of these environmentally acquired bacteria include soil-associated, plant-associated, and host-associated bacteria, such as *B. thuringiensis*, *Bacillus* spp., *Pseudomonas* spp., and Enterobacteriaceae, some with reported tick-pathogenic potential. This supports the idea that tick-pathogenic bacteria and, potentially, tick-colonizing bacteria can be isolated from ticks and tick microhabitats. Thus, the tick gut microbiota may hold the key to uncovering novel bacterial strains with tick toxicity and tick establishment potential. Furthermore, the establishment of a culturable bacterium in the tick gut that could persist through tick generations could be a promising tool for an indirect tick control strategy via manipulation of the established bacteria or paratransgenesis.

## Figures and Tables

**Table 1:** Non-endosymbiont (non-intracellular) bacterial genera and bacterial families reported in hard tick species in different studies. The summarized table includes only the overrepresented and environmentally associated taxa.

Tick Species	Bacterial Taxa (Genus, Genus and Species or Family)	Tick Stage/Feeding Status/Tissue	Method	Ref.
*Ixodes* *scapularis*	*Enterobacteriaceae*, *Mycobacterium*, *Sphingomonas*, *Pseudomonas*	Female and male/engorged and unfed/individual whole body Larva/unfed/individual whole body	Culture independent/16S rDNA Illumina MiSeq V3–V4 regions	[40]
*Ixodes* *scapularis*	*Pseudomonas*, *Brevibacterium*, *Bradyrhizobium*, *Phenylobacterium*, *Sphingomonas*, *Acinetobacter*	Male/unfed/individual, whole body	Culture independent/16S rDNA Illumina MiSeq V4 region	[39]
*Ixodes* *scapularis*	* *Bacillus thuringiensis*, *Bacillus* spp., *Pseudomonas diminuta*, *Corynebacterium*, *Pasteurella*	Female and male/partially-fed/individual whole body	Culture dependent	[53]
*Ixodes* *ricinus*	* *Bacillus*, *Mycobacterium*, *Staphylococcus epidermidis*, *Micrococcus luteus*, *Rhodococcus*, *Pseudomonas*, *Enterobacter*	Female/questing/individual midgut	Culture dependent and culture independent/16S rDNA Illumina MiSeq V3–V4 regions	[57]
*Amblyomma americanum*	*Bacillus thuringiensis*, *Pseudomonas*, *Micrococcus luetus*, *Staphylococcus epidermidis*, *Microbacterium*, *brevibacterium*, *Brevibacillus*	Female/questing/individual midgut	Culture dependent and culture independent/16S rDNA Illumina MiSeq V3–V4 regions	[25]
	*Sphingomonas*, *Pseudomonas*	Female and male/engorged and unfed/individual whole body Larva/unfed/individual whole body	Culture independent/16S rDNA Illumina MiSeq V3–V4 regions	[40]
	** Enterobacteriaceae, Bacillales, *Pseudomonas*, *Bacillus*	Male and female/questing/whole body-pooled Nymph/unfed/pool	Culture independent/16S rDNA 454 V3–V5	[58]
	**^†^** *Flavobacterium*, *Methylobacterium*, *Cloacibacterium*	Adult female and male/questing/individual whole body	Culture independent/16S rDNA Illumina MiSeq V3–V4 regions	[59]
	Rhizobiales, *Enterobacter*, *Klebsiella*, *Pantoea*, Pseudomonadales, Flavobacteriales	Female/engorged/individual whole body and Larvae/unfed/pooled sample	Culture independent/16S rDNA Eubacterial primers	[60]

* Example taxon of soil and plant origin. ** Example taxon of host origin. ^†^ Example taxon of fresh water and soil origin.

**Table 2:** Summary of *Bacillus thuringiensis* (*Bt*) toxicity to ticks using live cells, spores, or toxins. Studies on tick mortality induced by *Bt* live cells and spores.

Tick Species	Stage/Sex	*Bt* Strain	Concentration (CFU/mL)	Exposure Time/Method	Length of Bioassay	*n* (♂,♀)	Mortality (%)	Ref.
*Amblyomma americanum*	Unfed male and female **	kurstaki	1.5 × 10^7^	60 min daily for 3 days (♂), 60 min daily for 7 days (♀)/voluntary feeding	15	18 (8,10)	61.2	[29]
		israelensis	1.5 × 10^7^			18 (8,10)	44.5	
		morrisoni	1.0 × 10^7^			18 (8,10)	38.9	
	Unfed nymphs	kurstaki	1.8 × 10^7^	60 min daily for 7 days/voluntary feeding	7	10	30	
*Amblyomma americanum*	Unfed female	kurstaki	1.6 × 10^7^	60 min daily (3 days)/voluntary feeding	8	16	60.5	*This study*
		israelensis	1.6 × 10^7^	60 min daily (3 days)/voluntary feeding	8	16	20	
*Ixodes scapularis*	Engorged larvae	kurstaki	1 × 10^8^	30 s/immersion	15		96	[99]
*Ixodes ricinus*	Unfed/female	*Bt* QpB11 *	2.25 × 10^9^	1 time (length not specified)/immersion	15	130	30	[98]
		*Bt* KpC1 *	2.33 × 10^9^				20	
	Unfed/male	*Bt* QpB11 *	2.25 × 10^9^		15	130	80	
			2.25 × 10^6^				30	
		*Bt* KpC1 *	2.33 × 10^9^				80	
			2.33 × 10^6^				40	
*Dermacentor reticulatus*	Unfed/female	*Bt* PO14 *	2.88 × 10^8^		15	130	50	
		*Bt* OpQ3 *	1.48 × 10^7^				10	
			4.17 × 10^8^				40	
	Unfed/male	*Bt* PO13 *	2.88 × 10^8^		15	130	80	
			4.17 × 10^8^				70	
**Studies on tick mortality induced by *Bt* toxins or *Bt*-extracted proteins**								
**Tick species**	**Stage/sex**	***Bt* strain**	**Concentration (mg/mL)**	**Days of exposure**	**Length of bioassay**	***n* (♀)**	**Mortality (%)**	**Ref.**
*Rhipicephalus microplus*	Engorged/females	*Bt* GP138	1.25	60 s/immersion	20		95.8 ± 2.42	[95]
		*Bt* GP140					85.41 ± 8.6	
		*Bt* GP139					79.15 ± 12.5	
		*Bt* GP123					91.6 ± 0.00	
*Hyalomma dromedarii*	Engorged/males and females **	kurstaki *	10,000	1 time exposure (length not specified)/spraying	5	50 (25,25)	90.0	[96]
			5000			50 (25,25)	80.0	
		Israelensis *	10,000			50 (25,25)	80.0	
			5000			50 (25,25)	70.0	
		thuringiensis *	10,000			50 (25,25)	70.0	
			5000			50 (25,25)	63.3	
	Unfed/males and females **	kurstaki *	10,000			50 (25,25)	83.3	
			5000			50 (25,25)	63.3	
		israelensis *	10,000			50 (25,25)	76.7	
			5000			50 (25,25)	66.6	
		thuringiensis *	10,000			50 (25,25)	70.0	
			5000			50 (25,25)	63.3	
			2500			50 (25,25)	53.3	
			1250			50 (25,25)	33.3	
*Hyalomma* spp.	Adult/not specified	*Btcps*	3000	5 min/immersion	15 days	Not specified	15.0	[97]
			2500				89.0	
			2000				87.0	
			1500				75.0	
		*BtSCM*	3000		15 days		68.0	
			2500				67.0	
			2000				51.0	
			1500				54.0	

* Only unique treatments/concentrations with the highest mortalities are shown. ** Male and female tick mortality is shown as average.
